# The Impact of Farmers’ Strategic Behavior on the Spread of Animal Infectious Diseases

**DOI:** 10.1371/journal.pone.0157450

**Published:** 2016-06-14

**Authors:** Damian Tago, James K. Hammitt, Alban Thomas, Didier Raboisson

**Affiliations:** 1 CIRAD, UMR CMAEE, F-97170 Petit-Bourg, Guadeloupe, France; 2 INRA, UMR1309 CMAEE, F-34398, Montpellier, France; 3 Toulouse School of Economics – Recherche, INRA, Université Toulouse, 31000, Toulouse, France; 4 Department of Health Policy and Management and Center for Risk Analysis, Harvard University School of Public Health, Boston, Massachusetts, 02115, United States of America; 5 IHAP, Université de Toulouse, INRA, ENVT, Toulouse, France; Universidad de Zarazoga, SPAIN

## Abstract

One of the main strategies to control the spread of infectious animal diseases is the implementation of movement restrictions. This paper shows a loss in efficiency of the movement restriction policy (MRP) when behavioral responses of farmers are taken into account. Incorporating the strategic behavior of farmers in an epidemiologic model reveals that the MRP can trigger premature animal sales by farms at high risk of becoming infected that significantly reduce the efficacy of the policy. The results are validated in a parameterized network via Monte Carlo simulations and measures to mitigate the loss of efficiency of the MRP are discussed. Financial aid to farmers can be justified by public health concerns, not only for equity. This paper contributes to developing an interdisciplinary analytical framework regarding the expansion of infectious diseases combining economic and epidemiologic dimensions.

## Introduction

Movement restriction policies (MRPs) are one of the most popular strategies implemented within and between countries to fight the spread of infectious animal diseases in Europe and worldwide [1]. Their efficiency has been demonstrated in the field, often in combination with other measures. In Europe, following the Council Directive 91/119/EEC, whenever an infectious disease is detected three zones are delimited over which different restrictions on animal movements are implemented.

Scale-free networks [2], which characterize the structure of many real-world networks, provide a theoretical basis for designing strategies for controlling infectious diseases in cattle and other livestock. Applications have been developed recently for animal [3] and human diseases [4] and for viral computer infections [5].

The main characteristic of scale-free networks is the existence of highly connected nodes, i.e. with a degree that greatly exceeds the average such that the degree distribution (the frequency distribution of the number of connections of each node) is fat-tailed. These nodes are called hubs and their high connectivity is the main reason why a disease, technology, or fashion can spread much more quickly through a scale-free network than through a random network [6].

The idea behind control strategies such as the MRP and vaccination [7] is that removing infected nodes or immunizing susceptible ones are efficient mechanisms to fight the spread of a disease. It has been found that in fat-tailed degree distribution networks, random immunization (for example vaccinating a proportion of nodes chosen randomly) is not effective in stopping the spread of a disease since for many diseases immunization of 80%-100% of the population is required to halt epidemics [8]. On the other hand, targeted immunization of the most highly connected nodes (hubs) is very effective, but requires global information on the network [9]. Nevertheless, strategies that use only partial information have been found to efficiently reduce the spread of diseases [10].

New efforts regarding the traceability of infected animals, such as the construction of databases recording every movement of cattle in a country [11], make implementation of the MRP more efficient since they allow tracking the origin of an infection and improving the efficiency of emergency procedures.

The removal or isolation of infected nodes will prevent the disease from continuing to spread but this strategy is effective only under demanding conditions. For example, surveillance protocols must be sufficiently efficient that infections are detected almost immediately, or farmers must detect and quickly report the presence of an infectious disease. The time between contagiousness of an animal and its detection depends on the disease, and early detection may not occur when clinical signs are not easily visible. Even active-surveillance programs may not detect contagious animals quickly enough when tests are imperfect. Moreover, farmers may be slow to report possible infection, in particular when immobilization of their animals can be associated with very large costs [12].

A great advantage of the MRP is that it can be implemented to control an infectious disease as soon as the disease is detected, whereas strategies based on vaccination require time to develop, produce, distribute, and administer the vaccine to large populations.

The efficiency of the MRP also depends on the disease to be controlled. Even if detection and isolation of infected farms are perfectly efficient, if the disease is transmitted by insects or other vectors the MRP cannot by itself assure that the epidemic will be halted, as observed during the 2008 BTV-1 epizootic [13].

Studies of human-disease epidemics have recently begun to incorporate human reactions [14]. Most of these studies point to the existence of preventive responses that can reduce the spread of the infection, such as the voluntary use of vaccines or facemasks that reduce the risk of being infected [15]. In the field of animal diseases, the human factor has received less attention. However, the role of biosecurity measures on the spread of animal diseases has been analyzed taking into account the externalities and strategic behavior of farmers [16, 17]. Among English and Welsh cattle farmers, it has been shown that behavioral changes have an impact on the implementation of disease-control programs [18]. In Asian countries, misbehavior by farmers (lack of perfect compliance with regulation) and the emergence of underground poultry markets have been recognized as possible consequences of implementing control strategies [19], but their impacts have not been quantified.

In the model we propose, changes in farmers’ behavior induced by implementation of the MRP can make the policy ineffective under certain conditions. This is due to a mismatch between the time that the MRP is announced in a country (when an infectious disease is detected) and the time that immobilization is implemented in a specific region of that country. This result holds even under the assumption of perfect compliance by farmers (no misbehavior). It can be explained by reasoning similar to that of the so-called “green paradox”, which states that some environmental policies oriented to slow global warming act as an announced expropriation of fossil fuels, inducing the owners of these resources to accelerate extraction, which accelerates global warming [20]. Hence possible induced changes in farmers’ behavior should be considered when designing control strategies for infectious diseases.

The remainder of the paper is organized as follows. First, a susceptible-infected epidemiological model at the farm level is described to analyze diseases that can be transmitted through both the cattle trade network and the geographical network. Second, a two-period economic decision model is presented to understand how the MRP can change the strategic behavior of farmers located close to the restriction zone. The conditions that trigger anticipatory sales are established and the effect of the MRP on the spread of an infectious disease is analyzed, with and without anticipation effects. The results are presented, the implications for anticipatory sales are discussed, and a corrective mechanism is proposed. Finally the conclusions are presented.

## Materials and Methods

### The epidemiological model

National cattle trade networks have been characterized by previous studies, which find that they have a fat-tailed degree distribution [21, 22]. The degree of a node represents the number of connections (edges) that this node has with the rest of the network. In networks with fat-tailed degree distributions, highly connected nodes (hubs) are more frequent than in random networks; the distribution of degrees is heavy-tailed with power-law behavior of the form P(k) ~ *k*^−*φ*^ with 2 ≤ *φ* ≤ 3, where P(k) is the density function evaluated for degree “k”.

To study the spread of an infectious disease under different scenarios a fat-tailed degree distribution network is constructed following the characteristics of the French cattle trade network described by Rautureau et al. [21]. The trade network was constructed using the Complex Networks Package [23] with a power-law degree exponent φ = 2.15 [21].

To mimic the French cattle network (Table 1), the nodes were ranked by degree and the top 0.04% were classified as markets, the next 0.54% as dealers, and the remaining 99.42% as farms. Each group (farms, dealers, and markets) is characterized by a probability of selling derived from the French cattle network and modeled as a random variable with a uniform distribution in order to capture some of the heterogeneity (Table 2). A node is connected to the trade network in each period when it sells animals, which is simulated as the outcome of a Bernoulli trial using the corresponding probability of selling. Rewiring of the network takes place at each time period.

**Table 1:** Main characteristics of the cattle trade network.

	Rautureau (2011)	Model
Size (number of nodes)	244,097	10,000
Farms (number)	242,706	9,942
(% of total nodes)	99.43%	99.42%
Dealers (number)	1,315	54
(% of total nodes)	0.54%	0.54%
Markets (number)	76	4
(% of total nodes)	0.03%	0.04%
Type of network	Fat-tailed degree distribution	Fat-tailed degree distribution
*φ* (distribution of nodes)	2.15	2.15

**Table 2:** Calibration of the probability of selling.

	Rautureau (2011)	Model
	Weekly participation *	Probability of selling^#^
Active farms		
Min	32,920	13.6%
Max	58,605	24.1%
Active dealers		
Min	865	65.8%
Max	1,042	79.2%
Active markets		
Min	57	75.0%
Max	73	96.1%

* This variable represents the number of nodes that have at least one transaction for a specific week.

^#^ This probability is computed as the number of active farms (dealers or markets)/total number of farms (dealers or markets)

We extend the standard susceptible-infected (SI) epidemiological model to incorporate features that are essential for our analysis: the efficiency with which infectious disease is detected and the existence of multiple channels of infection. In our framework, each node represents an agent involved in the cattle trade (farm, dealer, or market) and each agent can be in one of two states: susceptible (*S*) or infected (*I*). If detection is imperfect, there are two types of infected nodes: detected (*I*_*d*_) and non-detected (*I*_*nd*_), so the total number of infected nodes is *I* = *I*_*d*_ + *I*_*nd*_.

To adapt the model to vector-borne diseases an additional transmission channel must be considered. With vector-borne diseases, the infection can propagate through (a) commercial flows and (b) geographical proximity to infected nodes. The incorporation of both channels has been highlighted as a desirable feature for realistic epidemiologic models [24]. An additional geographic network capturing the risk of vector-transmitted infection of nodes located close to infected ones has to be taken into account.

**Fig 1:**
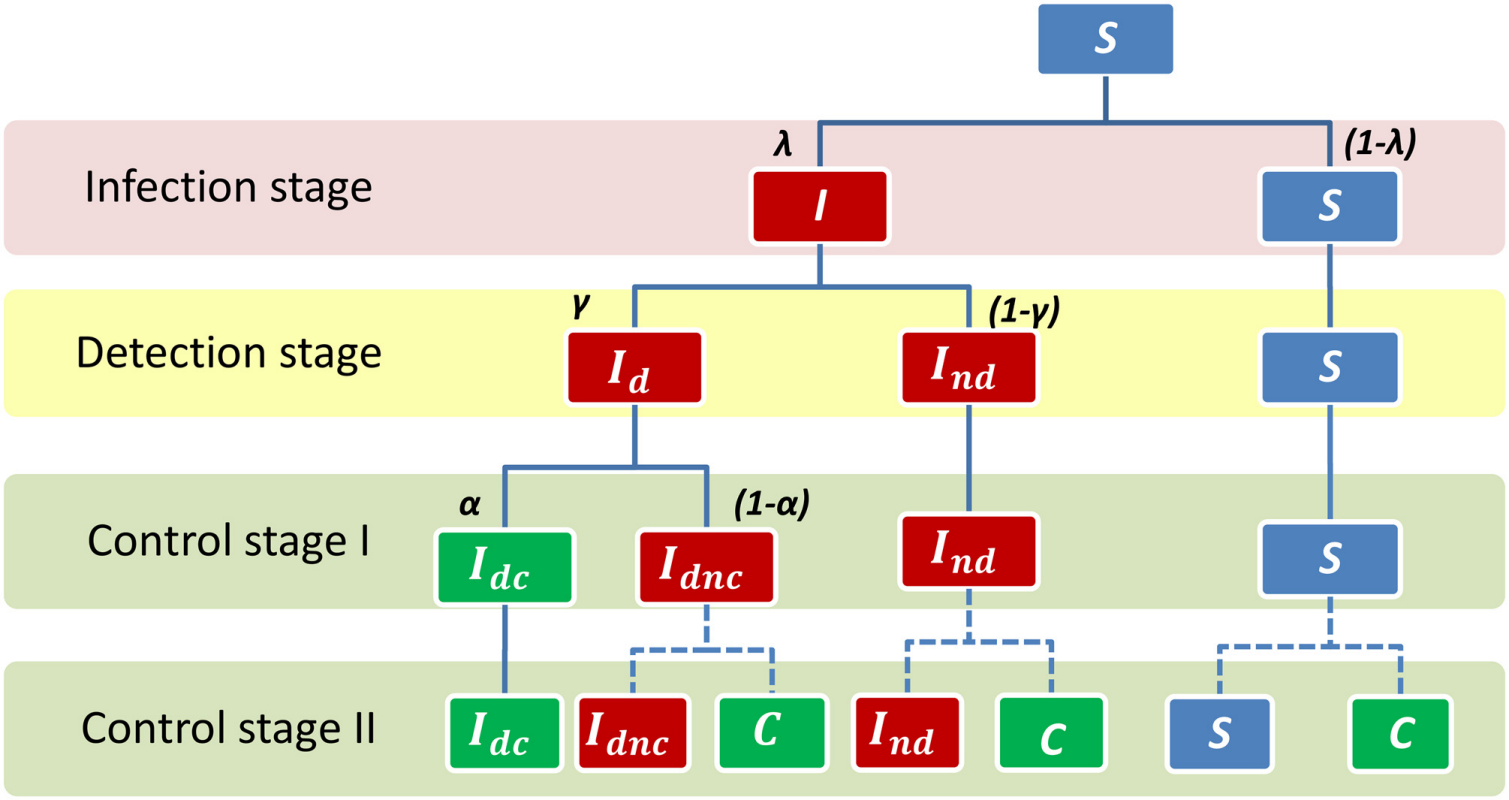
Tree diagram for a susceptible node. The diagram represents one time period. It starts with a susceptible node at risk of infection. At the infection stage, the node changes to infected status with probability λ; otherwise it retains the S-status during the whole period. At the detection stage, an infected node is detected with probability γ and adopts the I_d_-status; otherwise it takes the *I*_*nd*_-status. When disease is detected the node is removed from the network with probability α. Nodes with *I*_*nd*_-status and those with *I*_*d*_-status that are not removed (*I*_*dnc*_) remain in the network until the next time period when they are again at risk of being detected or removed. Nodes can be removed from the network if they are infected, detected, and effectively controlled (*I*_*dc*_ at control stage I), or if they enter a protection zone triggered when any direct neighbor in the geographic network is infected and controlled (*C* at control stage II).

An overview of the baseline model for a susceptible node exposed to infection is illustrated in Fig 1. At each time period there are 4 stages. In the first stage the trade of cattle takes place. At this stage all the market decisions are taken, animals are traded, and susceptible nodes can be exposed to infection through the arrival of infected animals. Second, there is the infection stage in which susceptible nodes exposed to infection (i.e. directly connected to an infectious node) can change from the *S*-state to the *I*-state with probability λ. The probability of a node becoming infected depends on the number of infectious neighbors and can be computed as 1-(1- λ)^N^, where N is the number of infectious neighbors. After the infection stage the disease is detected with probability γ, which is equal to 1 if detection is perfect. Finally the control stage takes place, in which movement-restriction policies are implemented in two steps: first, the *I*_*d*_-nodes are isolated with probability α; second, direct neighbors at the geographic network of infected and controlled nodes are isolated.

Table 3 summarizes the main parameters involved in the transition between states. The transmission rate λ depends on the disease and may also depend on other factors such as cattle density [25] and environmental factors [13], which affect vector capacity (ability of vectors to acquire and transmit pathogens). The detection rate γ depends on two factors: the characteristics of the disease and the efficiency of the detection tools. The former is associated with the length of the incubation period or the rate of subclinical cases while the latter may depend on the sensitivity of available tests. The parameter α reflects the efficiency of the authorities in implementing the MRP. Notice that the MRP isolates the nodes detected as infected as well as their geographic neighbors, which are considered to be those nodes directly linked through the geographic network. This simulates establishing a protection zone around the infected perimeter in which no infected animals have been detected but movements are restricted.

**Table 3:** State transitions and main parameters in the epidemiological model.

Event	Original state	Final state	Parameter	Description
Activation	NA	NA	*ps (Table 2)*	Bernoulli trial according to the probability of selling (ps) to decide if the node gets activated
Susceptible*	Not Susceptible	Susceptible	*NA*	A node is susceptible if it has an edge with an activated infected node (Geo and Trade networks)
Infection (*S*: *I*)	Susceptible	Infected	*λ = 5%*	Bernoulli trial according to the probability of infection (λ) to decide if the node gets infected
Detection (*I*: *Id*)	Infected	Detected	*γ* ϵ *{20%*, *50%*, *100%}*	Bernoulli trial according to the probability of detection (λ) to decide if the infected node is detected
Control A (*Id*: *Idc*)	Detected	Removed	*α = 100%*	Bernoulli trial according to the probability of control (α) to decide if the detected node is isolated from the network
Control B (*any state*: *C*)*	Any state	Removed	*NA*	A node is isolated from the network if connected to an "*Idc*-node" through the Geo network

* This event relies on the design of the network and there is no parameter directly involved

The geographic network is simplified to a square lattice in which each node is connected to its immediate neighbors, so there are no hubs in the network. Both networks have the same number of nodes and are associated through a one-to-one mapping that randomly assigns the node’s location, which remains fixed throughout the simulation. The infection is introduced by infecting one random node in the first period and the spread of the infection is summarized by the number of infected nodes up to 300 time periods. Since the probability of selling was derived for weekly cattle trade networks, a time period can be interpreted as a week in this analysis. For each scenario explored, 300 simulations were performed using Matlab (MathWorks).

### The economic model of anticipatory behavior

The immobilization of animals may impose costs on producers whose profits rely on the movement of their animals [26, 27]. To illustrate the implications of the MRP on the behavior of farmers, we consider a simple two-period economic model where a seller of cattle has to choose when to sell his animals.

Consider a risk neutral agent who has to choose between selling or not selling in each period (t = {1,2}). At any time period t, if the farmer decides to sell he receives:

$$Y_{t}=p_{t}*w_{t},$$ (1)

where: *Y*_*t*_ is the farmer’s income, *p*_*t*_ is the market price of cattle, and *w*_*t*_ is the total weight of the animals that the farmer sells in period t.

If the farmer decides to keep his animals and wait for the second period, *Y*_1_ = -c, where c represents the costs associated with keeping the animals for an additional period such as feeding and labor costs. To simplify the problem, assume there is no market risk or price trend such that *p*_1_ = *p*_2_ = *p*, and the animals gain weight according to the growth parameter *d* (i.e. *w*_2_ = *w*_1_ * (1 + *d*), where *d* represents the one-period weight gain percentage).

In case the farmer decides not to sell at t = 1 nor at t = 2, he receives an option value V, which is smaller than the revenue he would receive by selling at t = 2 (i.e. *V* < *p* * *w*_1_ * (1 + *d*)). In the context of beef weaned calves (young animals that are sold to fattening units), if animals become too heavy/old, farmers may face a lower price, which can be represented by a penalty *P>0* on the option value (*V* = *p* * *w*_1_ * (1 + *d*) * (1 − P)). This penalty does not represent fluctuations of market prices (which are fixed in our model) but the fact that if a farmer does not sell at the right moment, the animal losses some of the characteristics valued by buyers. For example, the cost of transporting beef weaned calves increases as the animals become heavier, which makes fattening units less prone to buy these animals.

The present study focuses on the most interesting case, where market conditions are such that the gain from waiting one period is larger than the cost of keeping the animals (i.e. *c* < *p* * *w*_1_ * *d*), making the strategy of waiting at t = 1 and selling at t = 2 strictly dominant. Usually, beef weaned calves producers face this type of situation and feeding costs play an important role in their decision.

If an infectious disease is detected at t = 1, a farmer sufficiently close to the infected zone will face the risk that the restricted zone (RZ) will expand to include his location by the next period (with probability q). If at t = 2 the farmer is located in the RZ he will not be able to sell. Hence a farmer that does not expect any compensation in case of falling into the RZ will decide to anticipate and sell prematurely if:

$$p\;*w_{1}>q\;*\;\left(V-c\right)+\left(1-q\right)\;*\;\left(p\;*\;w_{1}\;*\;\left(1+d\right)-c\right)$$ (2)

Solving for *q* yields

$$q>\frac{p*w_{1}*d-c}{p*w_{1}\left(1+d\right)-V}$$ (3)

If the gain from trading in the first period is larger than the payoff from keeping the animals and being unable to sell them in the second period (i.e., [*V* − *c*] < *p* * *w*_1_) the existence of *q* ∈ (0,1) is assured.

**Fig 2:**
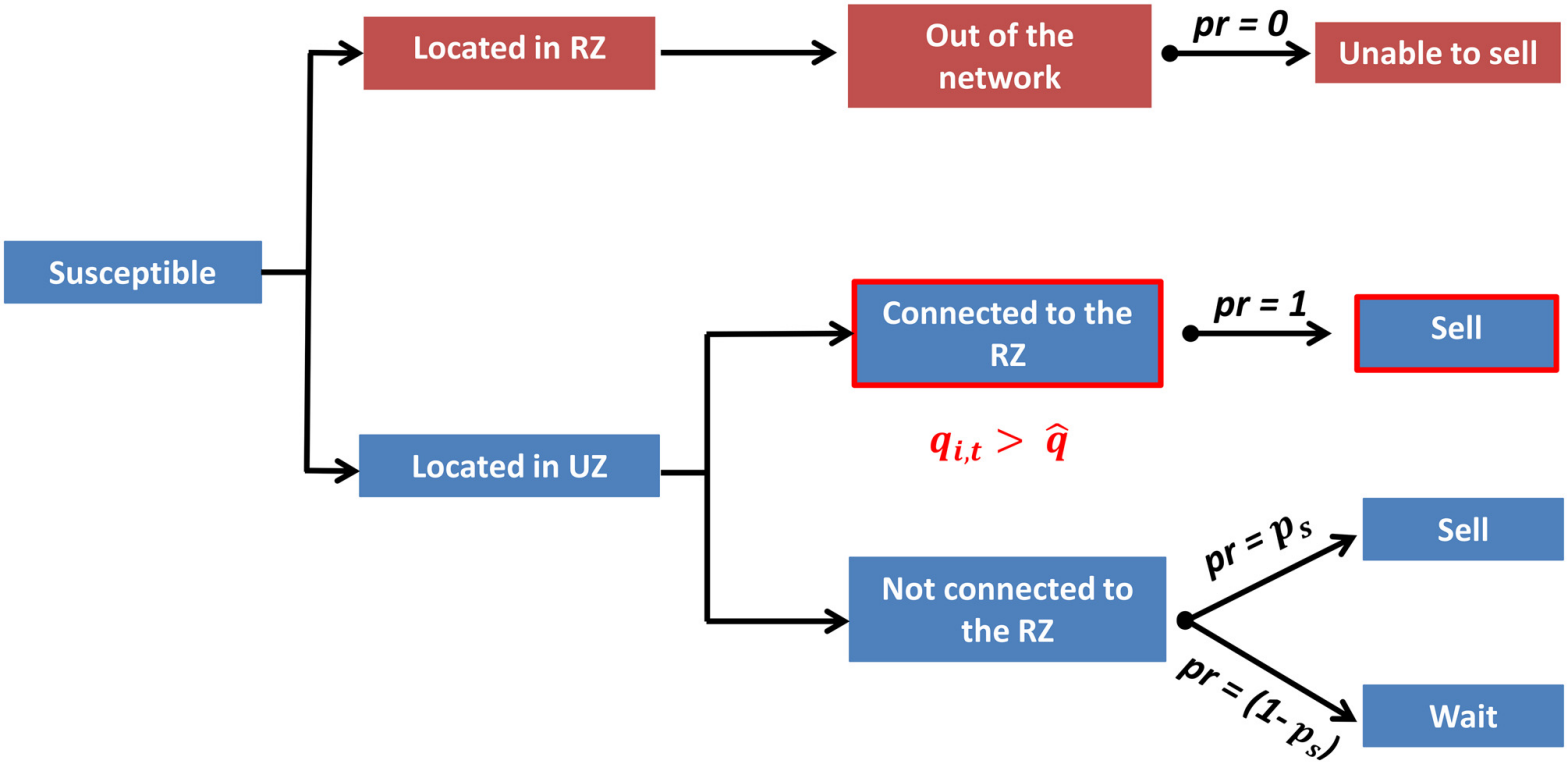
Anticipatory behavior—connectivity of a susceptible node. A susceptible node can be located in the RZ or the unscathed zone (UZ). If located in the RZ, the node is unable to sell (probability of selling = pr = 0) and has no effect on the dissemination of the disease. A node located in the UZ can face two situations: A) The node is not connected to any node located in the RZ so it will decide to sell or not according to pr = p_s_, which is associated with its type (farm, dealer, market; see Table 2); B) The node is connected to a node located in the RZ so ${\text{q}}_{\text{i},\text{t}}>\;\hat{\text{q}}$, which triggers anticipatory sales (pr = 1).

To incorporate the anticipatory behavior into the SI model, define *NR*_*it*_ as farm i’s set of neighbors in the geographic network that are located in the RZ at period t, and $\hat{q}\equiv \frac{p\;*\;w_{1}\;*\;d-c}{p\;*\;w_{1}\left(1+d\right)-V}$ as the threshold for *q* above which anticipatory selling is an optimal choice. We assume

$$\forall i\;such\;that\;NR_{it}\neq \emptyset ,\;q_{it}>\hat{q}$$ (4)

i.e. farms geographically adjacent to a farm in the RZ perceive the probability of being in the RZ in the next period to be high enough such that it is optimal to sell in anticipation (Fig 2). Therefore, anticipatory sales affect the rewiring process, which modifies the spread of the disease.

A monetary transfer to farmers in the RZ is a simple mechanism to avoid (or reduce) the anticipatory behavior of farmers at risk of entering the RZ. The size of the monetary transfer (denoted by T) that makes farmers with probability *q*_*it*_ of entering the RZ in the next period indifferent between anticipating and waiting can be derived from:

$$p\;*\;w_{1}=q_{it}\;*\;\left(V-c\;+\;T\right)\;+\;\left(1-q_{it}\right)\;*\;\left(p\;*\;w_{1}\;*\;\left(1\;+\;d\right)-c\right)$$ (5)

Therefore:

$$T_{it}=\{\begin{array}{ll} \;\left[\frac{p\;*\;w_{1}}{q_{it}}-\left(V-c\right)\right]-\left(\frac{1-q_{it}}{q_{it}}\right)\;*\;\left[p\;*\;w_{1}\;*\;\left(1\;+\;d\right)-c\right]\; & q_{it}>\hat{q}\\ 0\; & \;q_{it}\leq \hat{q} \end{array}$$ (6)

The size of the monetary transfer was calibrated taking information on prices, weight evolution of animals, and production costs, using data corresponding to the resurgence of the French BTV-8 epidemic in 2007. The monetary transfer is expressed as a function of the subjective probability of being immobilized in the next week. The main parameters of the economic model are summarized in S1 Table, including the values used to derive the monetary transfer required to avoid anticipatory sales.

The efficiency of the MRP was quantified by an index defined as the proportional increase in the time-weighted number of uninfected nodes associated with a policy, compared with no policy. This represents the proportional increase in the area above the curve of accumulated infected nodes due to the policy (S1 Fig). The index ranges from 0 to infinity, where 0 is associated with a policy with no benefits in terms of slowing the disease spreading.

A rank-correlation analysis was performed to explore how the characteristics and location of the initial case of infection contribute to variability in the results [28]. In this type of analysis, the degree of statistical dependence between two variables that follow different distributions is quantified and used to identify which factors are the most relevant to the spreading of a disease. The correlations explored are between the accumulated number of infected nodes at a specific time period (the period was chosen such that the average fraction of nodes infected was 50%) and three different variables that characterize the early periods of infection: the degree (number of edges) and closeness (defined as the inverse of farness, which is the sum of the distances from the selected node to the rest of the nodes in the network) of the initially infected node and the time period at which the first infection of a hub (dealer or market) takes place.

## Results

**Fig 3:**
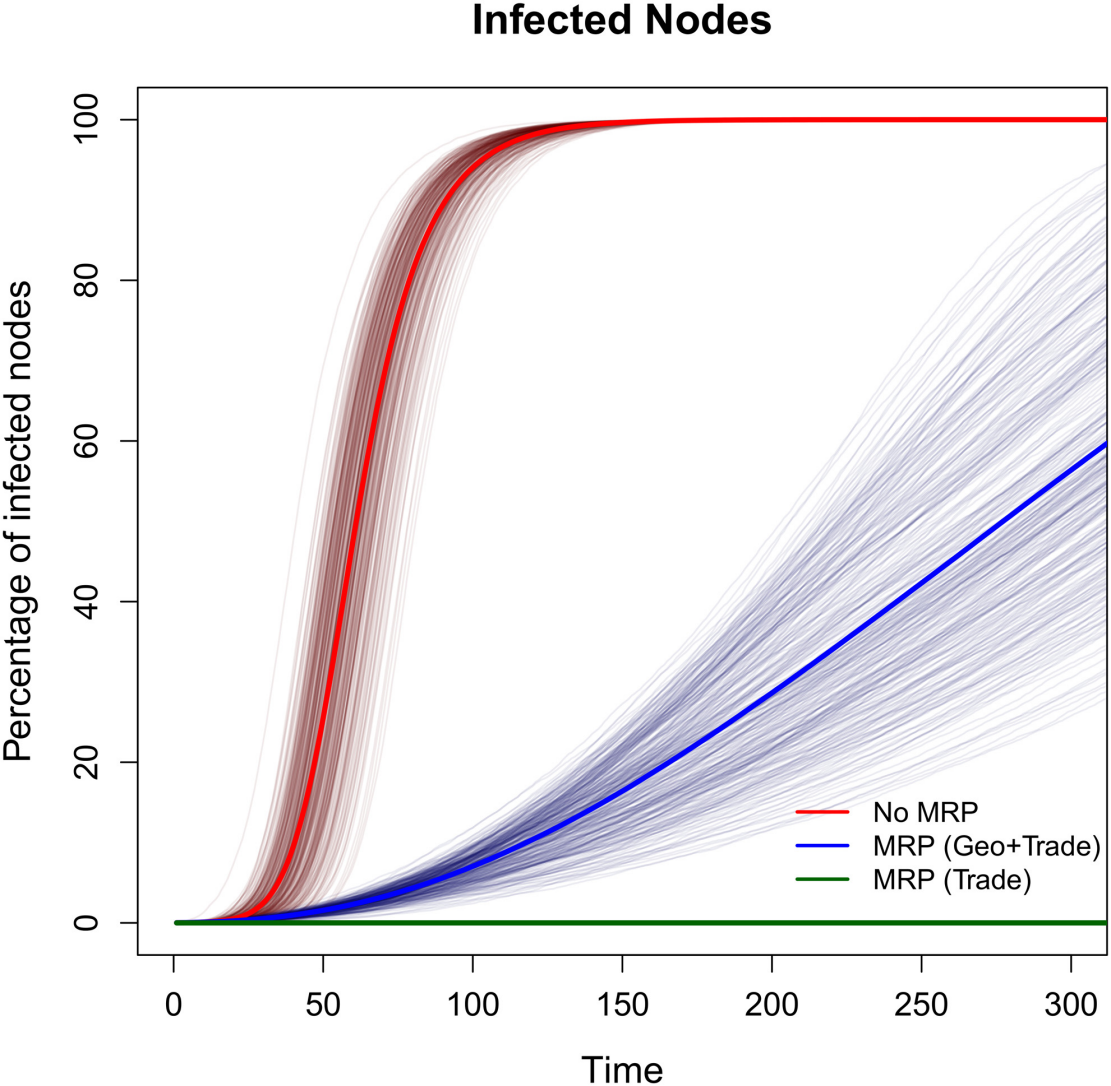
Effect of the MRP on the accumulated number of infected nodes. When the disease can be transmitted only through the trade network (green line), the MRP is a very efficient tool to stop the spreading of the disease. If the disease can be transmitted through the geographic network as well (blue lines), the MRP decreases the speed of infection but cannot contain it. These are the results over 300 simulations with: infection rate λ = 5%, detection rate γ = 100%, and control rate α = 100%. Average results are in thicker lines.

Results focus on the accumulated number of infected nodes over time after the introduction of a disease. In the absence of control strategies, a 5% infection rate leads to 100% of nodes being infected within approximately 100 weeks (Fig 3, red line). In the case of non-vector-borne diseases, i.e. when the transmission channel is restricted to the trade network, the MRP is an effective control strategy. If the detection tools are perfect it can immediately stop the spread of the disease (Fig 3, green line). In cases where the disease can be transmitted through both geographic and trade networks (as in the case of vector-borne diseases), the outbreak cannot be contained by movement restrictions alone even if the detection and control rates are 100% since the geographic channel cannot be closed by the MRP (Fig 3, blue line). However, the MRP helps to slow the disease spreading.

When a disease is difficult to detect (γ close to 0) the benefits of the MRP are severely dampened (S2 Fig). When both the geographic and trade networks are considered, a disease with a 5% infection rate infects half of the nodes around periods 275, 185, and 125 for detection rates equal to 100%, 50%, and 20%, respectively.

**Fig 4:**
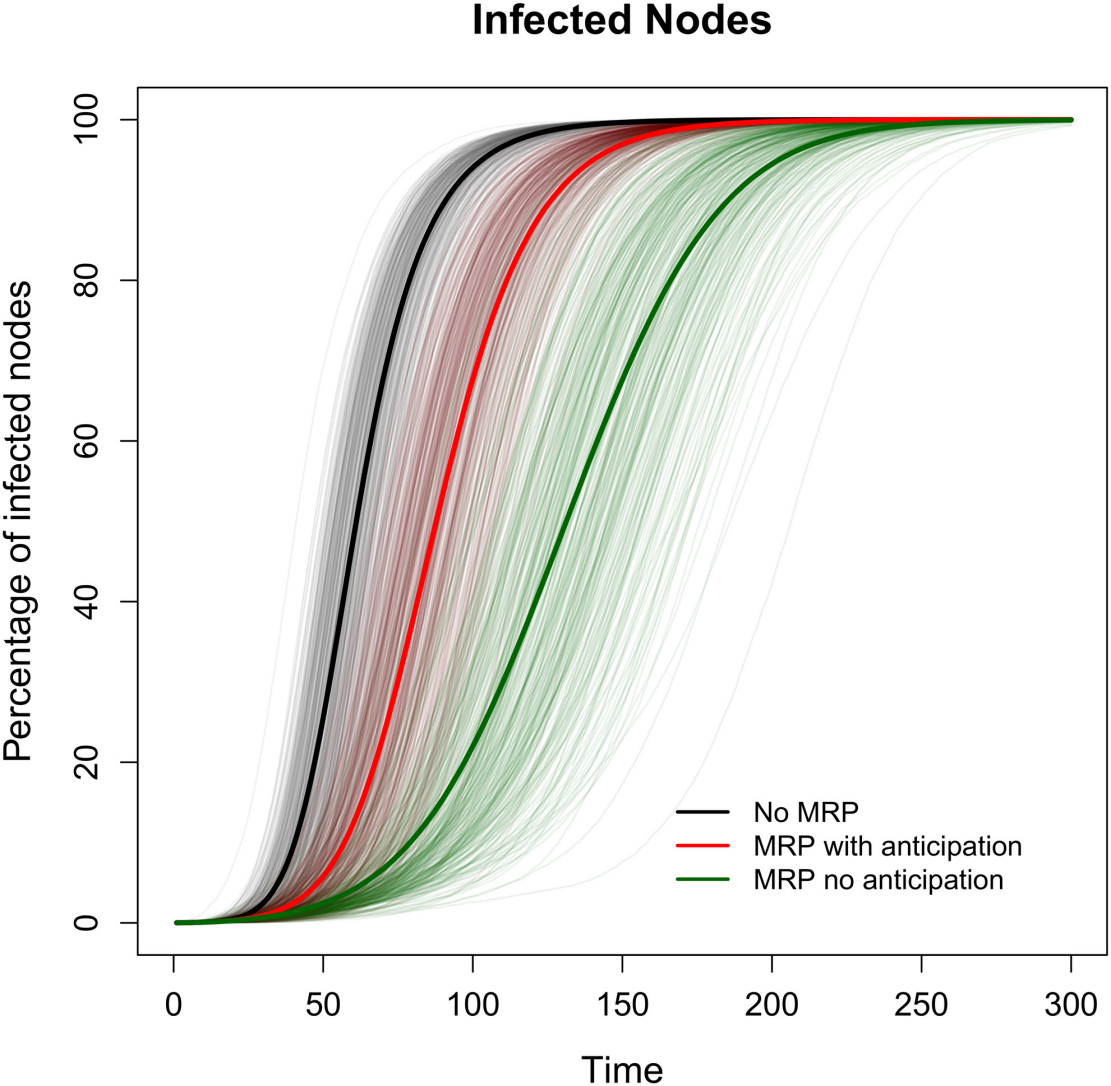
Accumulated infected nodes with and without anticipation effects. Comparison of the accumulated number of infected nodes for a disease transmitted through both the geographic and trade networks. The efficiency of the MRP decreases when anticipation effects are included. These are average results over 300 simulations with infection rate λ = 5%, detection rate γ = 20%, and control rate α = 100%.

When the strategic behavior of agents is incorporated the outcome of the MRP changes significantly. The anticipation effect increases the speed of infection, and for a low detection rate, γ = 20%, the benefits of the MRP are severely reduced (Fig 4). As the detection rate increases, the anticipation effects become less relevant since infected farms are removed from the network before they can sell.

**Fig 5:**
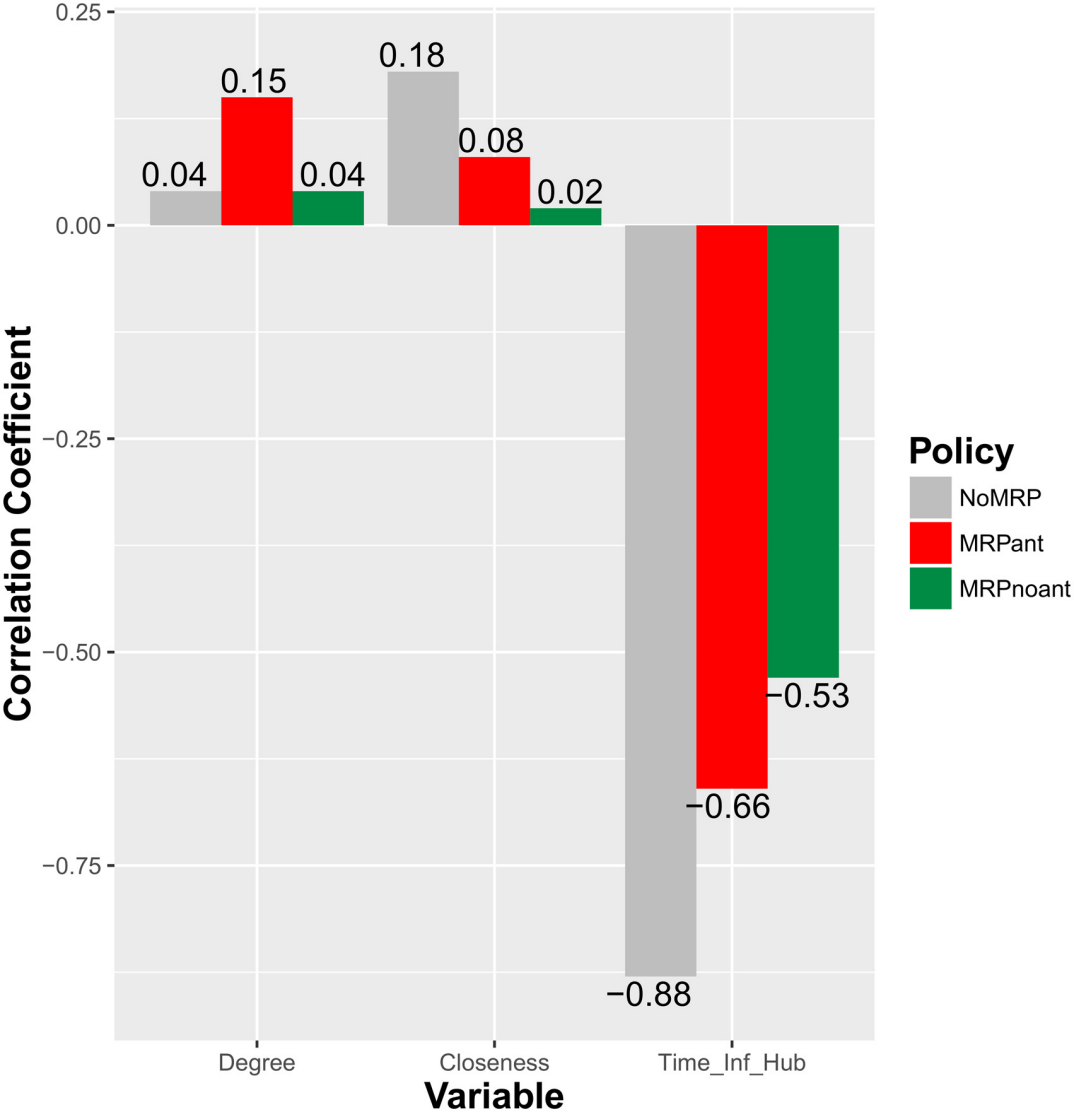
Rank correlation coefficient analysis for the number of infected nodes and the initial conditions of the epidemic. Due to the large differences between the outcomes under different scenarios, the number of infected nodes was computed for NoMRP at period t = 61, for MRP with anticipation (MRPant) at period t = 88, and for MRP with no anticipation (MRPnoant) at period t = 131. These periods correspond to timing at which 50% of the nodes were infected on the average trajectory.

Although there exists some overlap in the results of the simulations under different scenarios, the mean trajectory of infected nodes under the MRP with anticipation effects is significantly higher than in the case without anticipation. Such overlapping disappears when the 15% most extreme outcomes are omitted from the analysis (S3 Fig). The effect of anticipation can be assessed using the efficiency index, which decreases from 1.06 without anticipation effects to 0.41 when anticipation effects are included. The rank correlation coefficients, whose range is [–1,1], are small and positive for the degree and closeness of the initially infected node (between 0.02 and 0.18), and large and negative for the time at which the first hub is infected (between -0.53 and -0.88, see Fig 5). Finally, the size of the monetary transfer required to prevent anticipatory selling is derived for two option values that correspond to different penalties (10% and 30%) on the value of the animal, considering that too old/heavy animals are less desirable to fattening units. For probabilities above the threshold ($\hat{q}$) the monetary transfer is positive and increases at a decreasing rate (concave function) with an upper bound at *p* * *w*_1_ − (*V* − *c*) (S4 Fig).

## Discussion

The efficiency of the MRP is significantly reduced when facing a vector-borne disease because the policy cannot control the spread by vectors. However, the MRP restricts the transmission of the disease to the geographical channel. Because the geographic network has no hubs, the rate of spreading is significantly slower than through the commercial network. Geographic networks can be important even for diseases that are not spread by vectors. For example, it has been found that in the case of diseases transmitted through close animal-to-animal contact, such as foot-and-mouth disease, the plumes of the virus can be dispersed over long distances [29]. Therefore, considering both the geographic and trade networks can be important for some non-vector-borne diseases.

Factors that limit the speed of detecting infections are an obstacle for implementation of the MRP. The MRP becomes significantly less efficient when infected nodes that have not been detected spread the disease through both trade and geographic networks. The distribution of the time between the infection and control events has previously been highlighted as a crucial ingredient to identify for effectively managing disease spreading [30]. Factors that can limit the rate of detection include a high rate of subclinical cases, long incubation periods, and poor detection tools. The detection period can also be lengthened by the absence of active surveillance activities or lack of declaration in cases of clinical suspicion. For cow-calf systems or for dry dairy cows, farmers may easily miss some moderate clinical signs on some animals and not identify cases until clinical signs have become severe. Note that in the present study, changes in the efficiency of implementation of the control policy (α) have analogous results to changes in the detection rate (γ), since the effective removal of the infected nodes depends on the product α* γ.

The introduction of strategic behavior of farmers into the analysis significantly reduces the efficiency of the MRP. According to our efficiency index, the anticipatory behavior of farmers facilitates spreading of the disease and reduces the efficiency of the policy by 61%. When the MRP is implemented, farmers that perceive a high risk of entering the restriction zone will prefer to sell prematurely to avoid the immobilization costs. This increase in the connectivity of the network accelerates the dissemination of disease. In the case of vector-borne diseases, the holdings located close to the RZ have a higher risk of becoming infected. If the rate of subclinical cases is high or the incubation period is long, infection at farms outside the RZ may be unnoticed and may be transmitted through premature selling of undetected but infected animals. Although the MRP by itself is unable to stop the spreading of a vector-borne disease, it can be very useful for reducing the speed of spreading, especially when waiting for the availability of vaccines or for the seasonal inactivity of vectors.

The fat-tailed degree distribution of the cattle trade network plays an important role in accelerating the rate of disease spreading. Anticipatory sales can infect hubs, which can quickly infect many other nodes. If the trade network were homogenous, hubs would not exist and the effect of anticipatory sales on the spread of diseases would be smaller.

The assumption that all farmers having a neighbor (through the geographic network) in the RZ perceive that the risk of becoming immobilized in the next period is sufficiently high to induce them to sell their animals (Eq (4)) is strong. If this assumption is relaxed, there would be fewer anticipatory sales and the disease would spread more slowly.

Moreover, when an infected node is detected and isolated, its neighboring nodes are isolated too. Therefore, the way the restriction zone is defined has an impact on the results. If the MRP were modified to include not only direct neighbors but the neighbors of neighbors as well (2nd degree connections), the set of nodes that sell prematurely would change. In particular, at early stages of an epidemic, the number of nodes engaging in anticipatory selling (i.e. those at the frontier of the RZ) is increasing in the size of the RZ and decreases as the entire territory becomes RZ. But if there is a correlation between the risk of infection and the geographic proximity to infected nodes (as is the case of vector-borne diseases), by increasing the RZ, the nodes located at the frontier are less likely to be infected so they have a smaller effect on disease spreading even if they sell prematurely.

The rank-correlation analysis shows that the longer the hubs remain uninfected, the slower the epidemic will spread (Fig 5). This result is consistent with the recommendation of immunizing hubs as quickly as possible to avoid the expansion of a disease [31]. This relationship is stronger when no policy control takes place (R = -0.88) than when the MRP is implemented (R = -0.66 with anticipation, R = -0.53 without anticipation). On the one hand, the role of hubs as amplifiers of disease spreading is reduced through the MRP, but on the other hand, complementary strategies to control the spread of infection focused on the immunization of hubs will be less efficient compared to the situation without MRP. This trade-off between the benefits of implementing the MRP and the decrease in efficiency of complementary strategies (focused on the immunization of hubs) should be taken into account when designing an integrated control strategy.

The risk imposed by the anticipatory behavior is not a consequence of farmers violating the rules; anticipation effects can be important even in contexts where farmers report new infections promptly and comply with the movement restrictions. The key element in this model is that detection is imperfect (because the tools are limited or the latent or subclinical period of the disease is long) so a farm could sell infected animals without knowing it.

A monetary transfer conditional on entering the RZ is proposed as a simple mechanism to avoid the change in behavior of farmers affected by the policy. This monetary transfer must be credible and announced in advance so that farmers know of it when considering anticipatory selling. In practice, the monetary transfer can be directed to a specific type of farmer since not all farmers have incentives to sell prematurely. For example, the MRP affects farms specialized in dairy products to a lesser extent than farms specialized in the production of young beef weaned calves, which are sold to fattening units as intermediate products. Recognizing this fact, during the 2006–2008 bluetongue outbreak the French government provided financial aid only to farmers who generated at least 50% of their revenue by the sale of livestock (C-DGPEI/SDEPA/C2007-4027) and to the producers of young beef weaned calves (NS-DGPEI/SDEPA/N2008-4019).

The monetary transfer to farmers located in the RZ reduces the costs associated with being immobilized and therefore discourages premature sales. The size of the monetary transfer for risk-neutral farmers is given by Eq (6). When the risk of falling into the RZ exceeds a threshold, (*q>$\hat{q}$*), the monetary transfer that should be offered in the case of getting into the RZ is positive (it can be shown that the transfer is increasing and concave in q).

In Eq (6), the first element of the monetary transfer $\left(\left[\frac{p*w_{1}}{q_{it}}-\left(V-c\right)\right]\right)$ represents the gain from selling prematurely adjusted by the risk of falling into the RZ compared with waiting and being unable to sell in the second period. The second element ($\left(\frac{1-q_{it}}{q_{it}}\right)\;*\;\left[p\;*\;w_{1}\;*\;\left(1+d\right)-c\right]$) represents the discount due to the uncertainty of the event. This discount is given by the odds of not getting into the RZ in the next period ($\left(\frac{1-q_{it}}{q_{it}}\right)$) that multiplies the gain from selling in the 2^nd^ period ([*p* * *w*_1_ * (1 + d) − *c*]).

The discount goes to zero for farms with a high risk of getting in the RZ (*q*_*it*_ →1). In this case the government should offer full compensation if the farmer gets in the RZ to avoid the anticipation. The rationale for this discount is that the government benefits from the incentives of the seller to wait and sell in the second period (which is the dominant strategy) and therefore does not need to offer a transfer equal to the full cost of immobilization.

In the past the rationale for providing financial aid to farmers affected by the MRP was compensatory. When anticipation effects are a risk factor for spreading the disease, giving financial aid to farmers facing movement restrictions becomes an issue of public health and the arguments in favor of this measure are stronger.

Additional measures that help in the control of infectious diseases can be classified in three categories: measures oriented to reduce the transmission rate such as vaccination or the use of insecticides in the case of vector-borne diseases; measures oriented to increase the detection rate such as the adoption of new technologies (e.g., thermal scanners) and the implementation of preventive protocols; and measures oriented to detect high-risk zones to target surveillance.

The third type of measure is possible only with the collection of accurate data and the construction of reliable models. The European effort of constructing national databases to register all the cattle movements is a significant advance and could be extended to other species.

Network analysis of the spread of disease that incorporates human responses to policy provides new insight into the efficacy of alternative control measures. Extensions of this analysis to risk-averse agents and multiple periods should be useful.

The current study considers both geographic and commercial networks as potential channels of disease transmission and highlights the relevance of incorporating economic and behavioral elements to evaluate control strategies. For simplicity, we assumed the same transmission probability in both networks. In reality, the transmission probability can differ between transmission paths (geographic or trading) and can change over time. Efforts to identify temporal and spatial risk factors of disease spreading associated with the landscape and meteorological conditions should be included in the analysis.

## Conclusions

The MRP is the main strategy to control the spread of infectious diseases when vaccination is not available (as is the case for newly discovered serotypes of diseases). Arguments for implementing MRP are weaker for vector-borne than other diseases because this additional transmission channel reduces the MRP’s efficiency. However, it must be taken into consideration that even in the worst-case scenario (vector-borne disease with high rate of subclinical cases) the MRP helps to slow the spreading. This can be very useful in some contexts, such as when the period of inactivity of the vectors (cold months) is approaching, when a vaccine is in the process of development, or even when it is necessary to wait for the vaccines to be dispatched, injected, or boosted (when two injections are needed). For each disease, the costs and the benefits of the MRP should be analyzed in order to take the best possible decision.

The strategic behavior of agents can be an important element to consider when analyzing the efficiency of control strategies. The MRP produces incentives that may yield behavioral responses that reduce its effectiveness. We have shown how the strategic behavior of farmers can reduce the efficiency of MRP in controlling the spread of infection. This loss in efficiency should be considered in order to produce more-accurate estimates of the expected benefits of the MRP. The inclusion of complementary measures among emergency protocols related to infectious diseases can increase the efficacy of these policies. By including in the emergency protocols the financial aid for farmers in the RZ, farmers know in advance that they will be compensated in case of entering the RZ and the anticipatory behavior may be avoided.

## Supporting Information

S1 FigEfficiency index of control policies.The efficiency index is defined as the area above the curve with control policy (areas A+B) minus the area above the curve with no control policy (area A), over the area above the curve with no control policy (area A), i.e. $\frac{\left(A+B\right)-A}{A}=\frac{A+B}{A}-1$.(TIF)

S2 Fig Effect of the detection rate. Comparison of the accumulated number of infected nodes for a disease transmitted through both the geographic and trade networks. The efficiency of the MRP decreases along with the detection rate (γ). These are average results over 300 simulations with infection rate λ = 5% and control rate α = 100%.
(TIF)

S3 Fig Overlapping of extreme simulation results. Comparison of the accumulated number of infected nodes for a disease transmitted through both the geographic and trade networks and omitting the most extreme results of the simulations. These are average results over 300 simulations with infection rate λ = 5%, detection rate γ = 20%, and control rate α = 100%.
(TIF)

S4 Fig Monetary transfer to avoid anticipatory sales. The monetary transfer required to avoid anticipatory sales by farmers is a function of the subjective probability of being in the RZ in the next period (q) for different option values (V = p*w_1_*(1+d)*(1-P), where P is the penalty associated with less desirable animals). The model is calibrated using French data for August 30^th^, 2007 and estimates for the weight evolution of a Charolais calf: p = 2.56 eur/kglwt; w_1_ = 350 kg; d = 10/350; c = 8.73 eur.
(TIF)

S1 Table Parameters for the economic model. (DOCX)
