# Supplementary figures and images for: The Impact of Farmers’ Strategic Behavior on the Spread of Animal Infectious Diseases
Source: PLoS One. 2016 Jun 14;11(6):e0157450. doi: 10.1371/journal.pone.0157450 (PMC4907430; doi:10.1371/journal.pone.0157450)

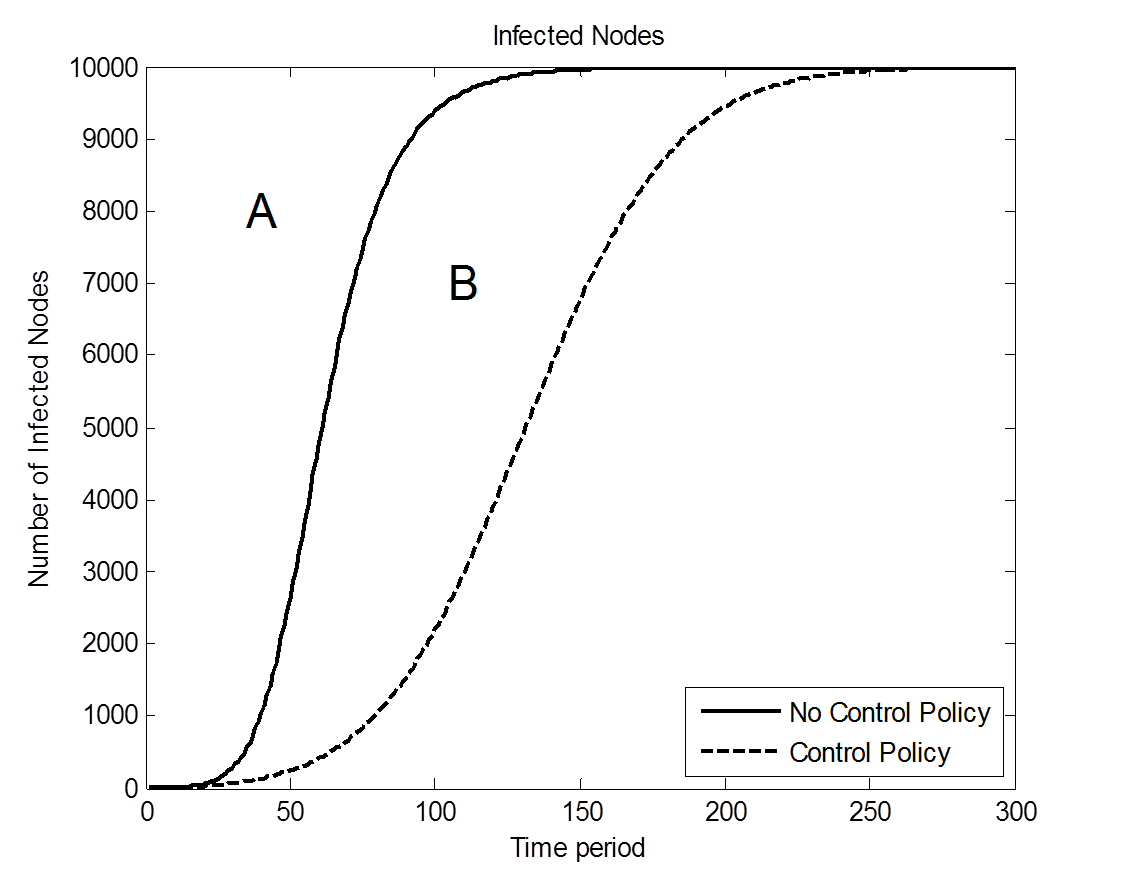

Supplement: S1 Fig — The efficiency index is defined as the area above the curve with control policy (areas A+B) minus the area above the curve with no control policy (area A), over the area above the curve with no control policy (area A), i.e. (A+B)−AA=A+BA−1. (TIF) [file pone.0157450.s001.tif]

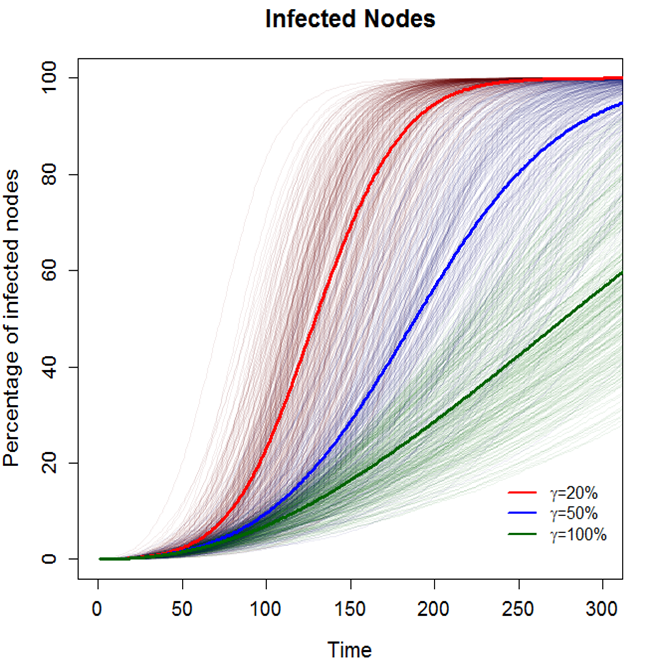

Supplement: S2 Fig — Comparison of the accumulated number of infected nodes for a disease transmitted through both the geographic and trade networks. The efficiency of the MRP decreases along with the detection rate (γ). These are average results over 300 simulations with infection rate λ = 5% and control rate α = 100%. (TIF) [file pone.0157450.s002.tif]

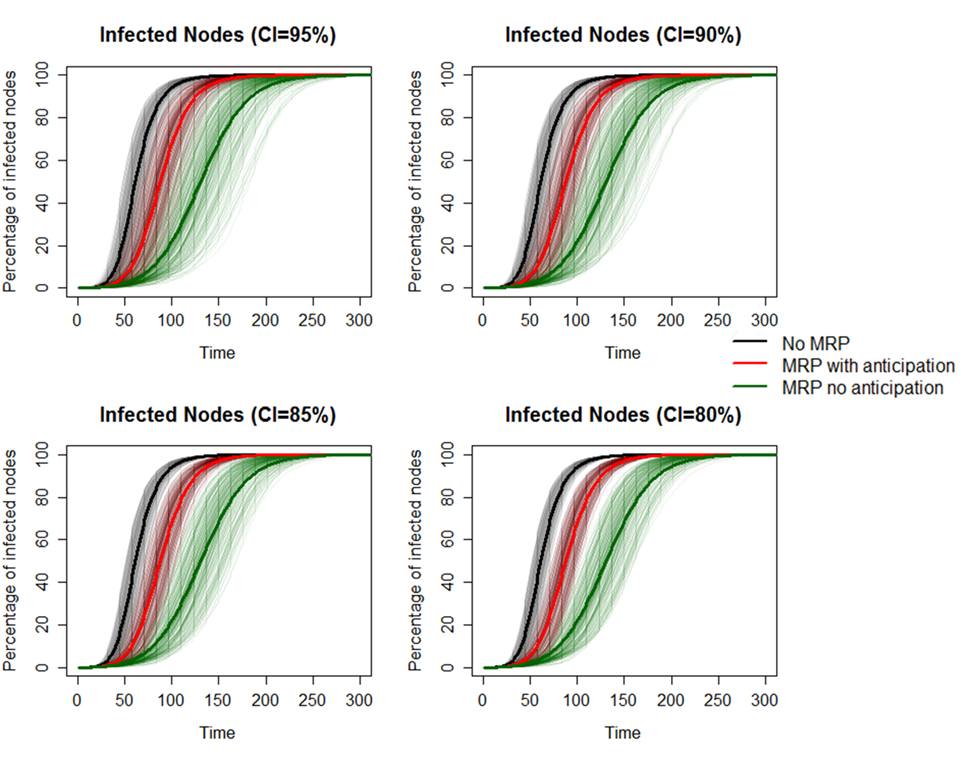

Supplement: S3 Fig — Comparison of the accumulated number of infected nodes for a disease transmitted through both the geographic and trade networks and omitting the most extreme results of the simulations. These are average results over 300 simulations with infection rate λ = 5%, detection rate γ = 20%, and control rate α = 100%. (TIF) [file pone.0157450.s003.tif]

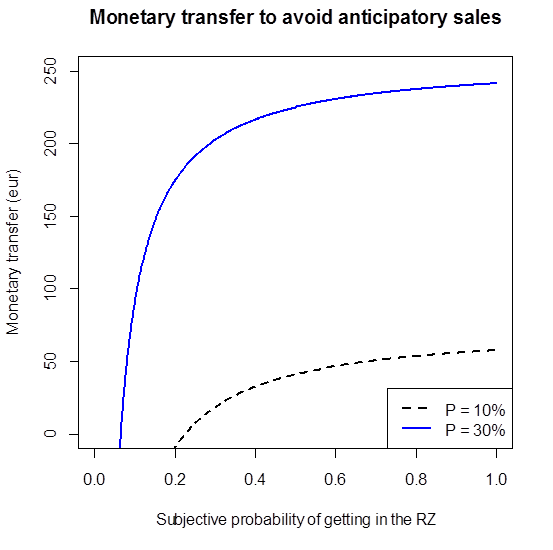

Supplement: S4 Fig — The monetary transfer required to avoid anticipatory sales by farmers is a function of the subjective probability of being in the RZ in the next period (q) for different option values (V = p*w1*(1+d)*(1-P), where P is the penalty associated with less desirable animals). The model is calibrated using French data for August 30th, 2007 and estimates for the weight evolution of a Charolais calf: p = 2.56 eur/kglwt; w1 = 350 kg; d = 10/350; c = 8.73 eur. (TIF) [file pone.0157450.s004.tif]
