# Supplementary material for: The Impact of Farmers’ Strategic Behavior on the Spread of Animal Infectious Diseases
Source: PLoS One. 2016 Jun 14;11(6):e0157450. doi: 10.1371/journal.pone.0157450 (PMC4907430; doi:10.1371/journal.pone.0157450)
Supplement: S1 Table — (DOCX) [file pone.0157450.s005.docx]

**S1 Table. Parameters for the economic model**

| **Variable** | **Meaning** | **Value for deriving the monetary transfer** |
| --- | --- | --- |
| Yt | Income at period t | pt * wt |
| pt | Market price of cattle at period t | 2.56 eur/kglwt^a^ |
| wt | Total weight of animals at period t | 350 kg |
| c | Cost associated with keeping the animals 1 period | 8. 73 eur |
| d | Weekly gain in weight of animals (as %) | 10/350 |
| V | Option value obtained if animals are not sold in last period | p * w1 * (1+d)*(1-P) |
| P | Penalty in the value of animals that are not sold in last period | {10%, 30%} |
| q | Perceived probability of getting in the RZ in the next period | [0,1] |

^a^ kglwt = kilogram of live weight
